# Active Virtual Reality for Chronic Primary Pain: Mixed Methods Randomized Pilot Study

**DOI:** 10.2196/38366

**Published:** 2022-07-13

**Authors:** Natalie Tuck, Catherine Pollard, Clinton Good, Caitlin Williams, Gwyn Lewis, Murray Hames, Tipu Aamir, Debbie Bean

**Affiliations:** 1 The Health and Rehabilitation Research Institute Auckland University of Technology Auckland New Zealand; 2 The Pain Management Unit, Department of Anaesthesiology and Perioperative Medicine Waitematā District Health Board Auckland New Zealand; 3 The Auckland Regional Pain Service Auckland District Health Board Auckland New Zealand

**Keywords:** chronic pain, virtual reality, VR, rehabilitation, serious games, physiotherapy, pain management, acceptability, intervention, feasibility

## Abstract

**Background:**

The modern management of chronic pain is largely focused on improving functional capacity (often despite ongoing pain) by using graded activation and exposure paradigms. However, many people with chronic pain find functional activation programs aversive, and dropout rates are high. Modern technologies such as virtual reality (VR) could provide a more enjoyable and less threatening way for people with chronic pain to engage in physical activity. Although VR has been successfully used for pain relief in acute and chronic pain settings, as well as to facilitate rehabilitation in conditions such as stroke and cerebral palsy, it is not known whether VR can also be used to improve functional outcomes in people with chronic pain.

**Objective:**

This study aimed to assess the feasibility of conducting an adequately powered randomized controlled trial (RCT) to test the efficacy of VR in a chronic pain treatment center and assess the acceptability of an active VR treatment program for patients in this setting.

**Methods:**

For this mixed methods pilot study, which was designed to test the feasibility and acceptability of the proposed study methods, 29 people seeking treatment for chronic pain were randomized to an active VR intervention or physiotherapy treatment as usual (TAU). The TAU group completed a 6-week waitlist before receiving standard treatment to act as a no-treatment control group. The VR intervention comprised twice-weekly immersive and embodied VR sessions using commercially available gaming software, which was selected to encourage movement. A total of 7 VR participants completed semistructured interviews to assess their perception of the intervention.

**Results:**

Of the 99 patients referred to physiotherapy, 53 (54%) were eligible, 29 (29%) enrolled, and 17 (17%) completed the trial, indicating that running an adequately powered RCT in this setting would not be feasible. Despite this, those in the VR group showed greater improvements in activity levels, pain intensity, and pain interference and reported greater treatment satisfaction and perceived improvement than both the waitlist and TAU groups. Relative effect sizes were larger when VR was compared with the waitlist (range small to very large) and smaller when VR was compared with TAU (range none to medium). The qualitative analysis produced the following three themes: VR is an enjoyable alternative to traditional physiotherapy, VR has functional and psychological benefits despite continued pain, and a well-designed VR setup is important.

**Conclusions:**

The active VR intervention in this study was highly acceptable to participants, produced favorable effects when compared with the waitlist, and showed similar outcomes as those of TAU. These findings suggest that a confirmatory RCT is warranted; however, substantial barriers to recruitment indicate that incentivizing participation and using a different treatment setting or running a multicenter trial are needed.

## Introduction

### Background

Chronic pain is a leading cause of disability worldwide and represents a significant burden to individuals, societies, and health care systems [1,2]. Virtual reality (VR) has been successfully used to supplement conventional care across diverse medical and rehabilitation settings, including stroke and cerebral palsy [3], and VR technologies are increasingly being used in pain treatment settings to provide pain relief and facilitate rehabilitation [4-6].

Within the field of pain, VR has been predominantly used in acute pain settings as a nonpharmacological approach to pain relief in people undergoing painful medical procedures such as needle insertion and burn care [7,8]. In addition, the past decade has seen growth in the use of VR in chronic pain settings, where studies have generally focused on using VR to provide immediate pain relief via distraction or relaxation [4,9-14]. These interventions typically involve participants being immersed in a pleasant and distracting setting in which they can interact with a simulated environment. For example, in a proof-of-principle study on 13 people with chronic pain, an immersive VR game designed to teach mindfulness-based stress reduction led to reductions in pain scores immediately after a 12-minute session [9]. In another study, 30 people with chronic pain played a VR game specifically designed for pain management, with results showing a reduction in pain scores during gameplay and from the pre- to posttreatment time points [12]. Similarly, a randomized controlled crossover study of 20 people with chronic pain found that an immersive VR game led to greater reductions in pain scores than the baseline and control conditions [10]. Despite representing a useful start, studies focusing on the application of VR for short-term pain relief have not assessed long-term outcomes. Although findings suggest that applications designed to be relaxing or distracting can provide immediate analgesia, whether this approach leads to sustained improvements in other pain outcomes is not known.

The successful management of chronic pain relies on maintaining or improving physical activity, often despite ongoing pain [15]. In addition to short-term pain relief, VR may be used as a tool to encourage healthy movement [16]. VR has been used to support functional rehabilitation for spinal cord injury [17], traumatic brain injury [18], Parkinson disease [19], stroke [20,21], and phantom limb pain [22]. In these contexts, VR has been used to help participants increase their range of movement using neuromodulation and exposure paradigms, and recent work has begun to explore whether these principles can also be applied to other chronic pain conditions. For example, in a well-designed feasibility study, 52 participants with chronic low back pain were randomized to either VR or no-treatment control [23]. The VR group played 15 minutes of virtual dodgeball, designed to gradually increase lumbar flexion for 3 consecutive days. The results indicated that the VR group had greater increases in lumbar flexion during gameplay, with participants reporting a strong positive response to the game. Although there were no between-group differences in pain and functional outcomes 4 to 6 days after treatment, the authors attributed this to the brevity of the intervention, and a randomized controlled trial (RCT) testing the efficacy of a 9-week VR intervention is currently in progress [24].

In related work exploring whether VR can be used in chronic pain settings to encourage activity, 2 recent RCTs compared VR with physiotherapy treatment as usual (TAU) among people with chronic neck pain [25,26]. Both studies were based on 44 participants and administered VR interventions comprising 8 training sessions over 4 weeks. One of the studies used a game designed specifically to improve neck function [25], whereas the other used commercially available games [26]. Both studies reported pain-relevant outcomes immediately and at follow-up, with both reporting that the VR group showed at least equivalence to TAU across outcomes [25,26].

These studies suggest that VR can be used to encourage healthy movement using conditioning and exposure paradigms; however, to date, studies have typically focused on specific pain sites using targeted exercises [24-27], often with specifically designed health applications [23,25,28]. Although specifically designed games are appropriate in some treatment settings [28], there are also circumstances where commercially available games have advantages, which may be true for chronic pain treatment settings where people tend to present with widespread pain or pain in multiple sites. In many cases, general function and activities of daily living are more important therapeutic targets than training specific joints or muscle groups [29,30]. In these instances, commercially available VR games may be useful for supporting sustained behavior change and adherence to physical activity more broadly [31,32].

Overall, it seems likely that commercially available, immersive, and embodied games that encourage full-body movement may be a less threatening and more enjoyable way for people with diverse pain conditions to improve their general function. However, the authors are only aware of 1 pilot study that has tested this possibility. In this study, 16 veterans with chronic pain participated in daily VR sessions using commercially available VR games over a 3-week period. The authors reported improvements in kinesiophobia, pain intensity, pain catastrophizing, and pain-specific functioning in some participants [33]; however, whether this approach is also suitable for patient groups in clinical settings has not been examined. Moreover, the perspectives of end users have not been adequately assessed [28], and perceptions of whether commercially available VR games are considered an acceptable treatment option among people seeking treatment for chronic pain are not known.

### Study Aims

This mixed methods pilot study aimed to test the planned methods and approach by identifying whether it would be feasible to conduct an active VR RCT in a hospital-based chronic pain treatment center and whether active VR treatment would be considered acceptable to patients in this setting. The criteria for feasibility were as follows: (1) 30 participants recruited within a 6-month time frame; (2) ≥70% retention rate; and (3) effect sizes (ESs) for primary outcomes (pain intensity and interference) of ≥0.5, indicating that a sample of approximately 60 participants per treatment arm would be sufficient to detect an effect [34]. The criteria for acceptability to patients were that (1) session rating scales for enjoyment are ≥6 on Likert scales ranging from 0 to 10; (2) treatment satisfaction and perceived improvement are ≥4 on Likert scales ranging from 0 to 7; (3) no serious adverse events or increases in pain specific to the VR treatment; (4) outcomes for VR are at least equivalent to TAU; and (5) interviews indicate that participants enjoy the VR treatment, find it beneficial, and believe that it is an acceptable and appropriate intervention for chronic pain rehabilitation.

## Methods

### Ethics Approval

Ethics approval was granted by the Health and Disability Ethics Committee (New Zealand Ministry of Health; HDEC ref 19/CEN/106), and locality approval was granted by the Auckland District Health Board in July 2019. The trial was registered with the Australian New Zealand Clinical Trials Registry (ACTRN12619001170112; universal trial number U1111-1234-0487). Eligible patients were given a written information sheet, an informed consent form, and instructions for accessing the study website. Those interested in participating were advised to access the study website where the information sheet and consent form were replicated, and informed consent was indicated by clicking a link at the end of the web-based consent form. This action opened the baseline questionnaire, and the completion of this questionnaire enrolled participants in the study. Eligible participants who expressed interest in the clinic but did not complete the baseline questionnaire were contacted via telephone to address any questions or concerns they may have had about the study. Participants were able to complete informed consent procedures and baseline questionnaires in the clinic in a separate appointment if they preferred.

### Study Design

A mixed methods randomized pilot study was conducted with assessment points before and after 3 treatment arms to assess the feasibility and acceptability of a planned RCT, comparing an active VR intervention with a waitlist control and physiotherapy TAU.

### Participants

The study took place at a hospital-based interdisciplinary chronic pain center, The Auckland Regional Pain Service (TARPS), Auckland District Health Board, New Zealand. Patients attending TARPS and referred to physiotherapy between October 1, 2019, and November 30, 2020, were invited to participate. Inclusion criteria were having musculoskeletal pain, being aged 18 to 70 years, and being able to communicate in English. Participants were excluded if they had a severe medical or psychiatric condition, were receiving treatment for pain elsewhere, or if their health care was being funded by the Accident Compensation Corporation (New Zealand’s accident compensation provider).

### Procedures

#### Overview

Participants completed baseline questionnaires before being randomized using a web-based random number generator to either VR or waitlist followed by TAU. All participants wore activPAL activity monitors (PAL Technologies Ltd) and completed questionnaires at the start and end of each 6-week treatment period. Participants in the VR group completed session evaluations at each appointment, and 7 attended the semistructured interviews.

#### VR Intervention (Experimental Group)

Participants attended twice-weekly VR appointments for 6 weeks, supervised by a physiotherapist with 4 years of experience in using VR for chronic pain. The HTC Vive immersive VR system (HTC Corporation) was used with a head-mounted display and accompanying hand sensors. The VR software programs were run via a wall-mounted desktop display that allowed the physiotherapist to view the participant’s visual field. Games that encouraged full-body movements were selected, and participants were guided to perform physically active tasks within the virtual environment and progressed through VR games at the discretion of the treating physiotherapist (Multimedia Appendix 1).

#### Waitlist

Patients assigned to TAU completed a 6-week no-intervention waitlist to act as a no-treatment control before receiving standard physiotherapy treatment. Outcome measures were completed before and after the waitlist period.

#### TAU Group

Following the 6-week waitlist, the participants attended 6 weeks of physiotherapy treatment with 1 of 2 physiotherapists, each with >10 years of experience working in this chronic pain treatment setting. Therapy included education related to pain neuroscience, fear avoidance, and deconditioning, and participants were given home-based exercise regimens and tailored gym-based activity programs focused on graded activation and exposure therapy.

### Measures

#### Brief Pain Inventory

The Brief Pain Inventory (BPI) [35,36] measures pain intensity and interference on 11-point Likert scales. The BPI has adequate reliability and validity in populations with chronic pain [37] and is a recommended outcome measure for chronic pain clinical trials [38]. A 2-point reduction is considered the minimal clinically important difference (MCID) in pain intensity scores [38,39].

#### Kinesiophobia

The Tampa Scale of Kinesiophobia-13 (TSK-13) is a measure of fear of movement, injury, and reinjury [40,41]. Participants respond to items on 4-point Likert scales, with scores ranging from 13 to 52, with higher scores indicating greater fear of movement and (re)injury. The TSK-13 has sufficient reliability and validity in samples with chronic pain [42,43]. In this study, Cronbach α was .72, indicating adequate internal consistency. An 18% reduction in TSK-13 scores is considered an MCID [44].

#### Activity

Daily activity and step counts were collected using activPAL activity monitors during weeks 1 and 6 of VR, waitlist, and TAU. ActivPAL monitors have been validated in samples with chronic pain [45-46], and the average daily number of steps and activities (total minutes spent standing, walking, and cycling) were extracted.

#### Global Impression of Change

The Patient Global Impression of Change scale [47] is a single-item recommended pain outcome measure [38] of perceived improvement with treatment on a 7-point Likert scale ranging from 1 (*not at all improved*) to 7 (*very much improved*). Participants also indicated their satisfaction with treatment on a scale from 1 (*not at all satisfied*) to 7 (*very satisfied*).

#### Session Evaluation Questionnaire

Participants completed questionnaires at each VR treatment session to assess changes in pain intensity from the pre- to postsession time points, as well as the degree to which they found the VR session enjoyable and immersive, using 10-point Likert scales.

#### Semistructured Interviews

The qualitative component of this study was designed in accordance with the Critical Appraisal Skills Program (CASP) guidelines [48]. The first 7 participants to complete 8 VR treatment sessions attended 30 to 60-minute semistructured interviews designed to assess their expectations and perceived usefulness of the VR intervention, changes in pain and function, and suggestions for improvement.

### Data Analysis

Quantitative data were analyzed using SPSS (version 27). Baseline demographics and clinical characteristics were summarized using descriptive statistics (Table 1). Recruitment and retention rates, pain outcomes, enjoyment, and immersion scores were calculated. As this was a feasibility study, significance testing was not conducted. Instead, Hedges g relative ESs were used to estimate the necessary sample sizes for an RCT using G*Power software, with an α of .05 and power of 0.80. Ratings of pain, enjoyment, and immersion collected at the end of each VR session were averaged across the total number of attended sessions to create mean pain change, enjoyment, and immersion scores.

For qualitative data, interviews were transcribed and analyzed using reflexive thematic analysis [49,50] by a member of the research team (CW), with support from 2 additional team members (NT and DB). The 5 steps of reflexive thematic analysis were followed, where phase 1 (familiarization) involved reading and rereading the data while taking notes on features of interest. In phase 2, data were coded by applying brief unique labels to all quotes that appeared relevant to the research question. For phase 3, initial themes were generated by grouping quotes with similar codes and examining the resulting sets of quotes. In phase 4, 3 themes were developed and reviewed by looking at how each code fit with the proposed theme, the data supporting each code and theme, and the degree to which each theme helped answer the research question. Finally, in phase 5, 3 themes were named and defined. As noted earlier, qualitative analysis was conducted primarily by CW, a female physiotherapy student with training in reflexive thematic analysis. Qualitative analysis was supported by NT and DB, both of whom have PhDs in health psychology and prior experience working in an interdisciplinary pain center. Both NT and DB currently hold positions as senior research fellows, with training in reflexive thematic analysis.

**Table 1:** Demographic and baseline clinical characteristics (N=20).

Characteristics			Total (n=20)		Treatment		
					VR^a^ (n=10)		Waitlist and TAU^b^ (n=10)
Age (years), mean (SD)			40.1 (16.2)		41.3 (17.7)		38.7 (15.3)
Sex (female), n (%)			13 (65)		8 (80)		5 (50)
**Ethnicity, n (%)**							
	New Zealand European	12 (60)		6 (60)		6 (60)	
	Māori	2 (10)		1 (10)		1 (10)	
	Indian	3 (15)		2 (20)		1 (10)	
	Fijian	1 (5)		0 (0)		1 (10)	
	Other European	2 (10)		1 (10)		1 (10)	
**Employment,** **n (%)**							
	Full-time	5 (25)		2 (20)		3 (30)	
	Part-time	3 (15)		2 (20)		1 (10)	
	Retired	1 (5)		1 (10)		0 (0)	
	Unemployed	9 (45)		3 (30)		6 (60)	
	Student	2 (10)		2 (20)		0 (0)	
**Pain duration, n (%)**							
	<12 months	0 (0)		0 (0)		0 (0)	
	12-24 months	3 (15)		2 (20)		1 (10)	
	2-5 years	5 (25)		1 (10)		4 (40)	
	>5 years	12 (60)		7 (70)		5 (50)	
**Main pain location, n (%)**							
	Neck	2 (10)		0 (0)		2 (20)	
	Back	8 (40)		4 (40)		4 (40)	
	Stomach	2 (10)		1 (10)		1 (10)	
	Chest	1 (5)		1 (10)		0 (0)	
	Hips	1 (5)		1 (10)		0 (0)	
	Pelvis or groin	1 (5)		1 (10)		0 (0)	
	Knee	3 (15)		1 (10)		2 (20)	
	Leg	1 (5)		1 (10)		0 (0)	
	Foot	1 (5)		0 (0)		1 (10)	
BPI^c^ intensity, mean (SD)			8.3 (1.5)		8.4 (1.8)		8.1 (1.2)
BPI interference, mean (SD)			7.3 (1.6)		7.5 (1.7)		7.1 (1.5)
TSK-13^d^, mean (SD)			33.5 (5.4)		32.3 (5.4)		34.6 (5.4)

^a^VR: virtual reality.

^b^TAU: treatment as usual.

^c^BPI: Brief Pain Inventory.

^d^TSK-13: Tampa Scale of Kinesiophobia-13.

## Results

### Quantitative Results

#### Recruitment and Retention

Between October 1, 2019, and November 30, 2020, a total of 99 non–Accident Compensation Corporation patients were assessed at TARPS and referred to physiotherapy. Of these 99 patients, 53 (54%) met the inclusion criteria, and 29 (29%) were enrolled in the study. Of the 29 patients, 13 withdrew (n=5 from the VR group, and n = 4 from each of the waitlist and TAU groups), resulting in a final sample of 20 participants, of whom 10 were in the VR group, 10 in the waitlist group, and 6 in the TAU group. The recruitment rate of 2 participants per month, as well as the dropout rate of 45%, indicated that it would not be feasible to recruit approximately 60 participants per trial arm in this clinical setting.

#### Intervention Parameters

All participants in the VR arm completed ≥7 appointments, and 30% (3/10) completed 12 appointments. There were no adverse events or increases in pain specific to the VR intervention. Immersion scores ranged from 8.4 to 9.6, and enjoyment scores ranged from 8.0 to 9.9, thereby meeting the acceptability criteria.

#### Pain Intensity and Interference (BPI)

There were medium ESs favoring VR over the waitlist for a reduction in BPI pain intensity (ES=0.52) and BPI pain interference (ES=0.50). This suggests that for an α of .05, and a power of 0.80, a sample of 60 and 64 per group would be sufficient to detect significant effects for pain intensity and interference, respectively (Table 2).

**Table 2:** Mean change scores and relative effect sizes for pain-relevant outcomes, perceived improvement, and satisfaction with treatment.

Outcomes	Waitlist (n=10)		TAU^a^ (n=6)		VR^b^ (n=10)		VR versus waitlist, effect size (95% CI)	VR versus TAU, effect size (95% CI)
	Participants, n (%)^c^	Values, mean (SD)	Participants, n (%)^c^	Values, mean (SD)	Participants, n (%)^c^	Values, mean (SD)		
ΔBPI^d^ intensity	10 (100)	–0.30 (1.57)	6 (100)	–0.17 (2.32)	9 (90)	–1.00 (0.87)	–0.52 (–1.39 to 0.67)	–0.49 (–1.47 to 0.51)
ΔBPI interference	10 (100)	–1.10 (2.13)	6 (100)	–1.00 (1.39)	9 (90)	–2.06 (1.46)	–0.50 (–1.37 to 0.39)	–0.70 (–1.70 to 0.32)
ΔTSK-13^e^	10 (100)	–2.90 (5.17)	6 (100)	–4.00 (4.56)	9 (90)	–1.56 (5.57)	0.24 (–0.63 to 1.10)	0.44 (–0.55 to 1.42)
Change in daily steps	7 (70)	212 (2394)	6 (100)	1127 (2784)	8 (80)	852 (2934)	0.22 (–0.74 to 1.18)	–0.09 (–1.08 to 0.90)
Change in daily activity (minutes)	7 (70)	2.15 (59.03)	6 (100)	–21.05 (91.49)	8 (80)	19.45 (64.50)	0.26 (–0.70 to 1.22)	0.49 (–0.52 to 1.49)
Satisfaction (score: range 1-7)	9 (90)	4.78 (1.20)	6 (100)	5.83 (0.98)	9 (90)	6.11 (0.93)	1.18 (0.20 to 2.14)	0.28 (–0.71 to 1.25)
Improvement (score: range 1-7)	9 (90)	4.78 (0.83)	6 (100)	5.67 (1.03)	9 (90)	5.89 (0.78)	1.31 (0.31 to 2.28)	0.24 (–0.75 to 1.21)

^a^TAU: treatment as usual.

^b^VR: virtual reality.

^c^Number of valid participants with complete data.

^d^BPI: Brief Pain Inventory.

^e^TSK-13: Tampa Scale of Kinesiophobia-13.

#### Kinesiophobia

Small ESs favored the waitlist over VR (ES=0.24) and TAU over VR (ES=0.44). None of the groups met the criteria for MCID in kinesiophobia change scores (≥18% reduction; Table 2) [44].

#### Step Counts and Activity (Minutes Spent Standing, Walking, and Cycling)

There were small ESs favoring VR over the waitlist for both step count (ES=0.22) and activity scores (ES=0.26). Both VR and TAU met the criteria for an MCID in daily step counts (≥600-1100 step increase; Table 2) [51].

#### Treatment Satisfaction and Global Impression of Change

There were very large ESs favoring VR over the waitlist for treatment satisfaction (ES=1.18) and perceived improvement (ES=1.31) and small ESs favoring VR over TAU for treatment satisfaction (ES=0.28) and perceived improvement (ES=0.24; Table 2).

### Qualitative Results

Analysis of the transcribed interviews generated three themes: (1) VR is an enjoyable alternative to traditional physiotherapy, (2) VR leads to functional and psychological benefits despite continued pain, and (3) the importance of a well-designed VR setup.

#### Theme 1: VR Is an Enjoyable Alternative to Traditional Physiotherapy

Participants were enthusiastic about trying VR rehabilitation and had positive expectations of treatment; for example, one of the participants said the following:

I had really high hopes...I thought it might actually take my pain away

Male, 51 years, Fijian-Indian, chest and shoulder pain

Participants also described VR as more enjoyable, accessible, and achievable than their previous experiences of physiotherapy and described VR as *fun* and *exciting*:

It’s a really good way to incorporate fun activity into your life on a regular basis. And for someone who struggles to find the mental and physical energy to do anything like that, it’s a really good pull to get you up.

Female, 25 years, European, back pain

Participants also described VR as improving activity levels by distracting them from pain. Several participants described VR as *brain training* and believed that taking part in the VR treatment changed the way that their nervous system processed pain:

By doing a movement and not having that immediate thought that it’s going to hurt, it’s already training my brain so that it doesn’t go—nope you’re not doing that!—and then sending pain signals everywhere.

Female, 23 years, European, pelvic pain

Despite the perceived benefits, some participants expressed uncertainty about whether VR could be considered a legitimate treatment. They explained that the enjoyable nature of VR was inconsistent with their expectations of physiotherapy:

With VR you don’t really feel like it’s treatment...you have an expectation that when you go to [physical therapy] they give you exercises or manual treatment so when you leave it feels like you’ve done something. But when you’re doing VR, I don’t feel like I’ve necessarily achieved any treatment.

Female, 21 years, European, hip pain

Overall, the participants had positive expectations about VR, the rationale for VR treatment was clear to them, and they found VR to be highly agreeable. For some participants, the enjoyable nature of VR meant that it did not feel like a legitimate treatment approach.

#### Theme 2: Functional and Psychological Benefits Despite Continued Pain

Participants said that they experienced pain relief during the VR sessions; however, this was not sustained after treatment. Despite ongoing pain, participants reported an overall increase in daily physical activity, improved strength, reduced stiffness, improvements in sitting and standing tolerances, and greater confidence in engaging in activities of daily living. Participants said that VR changed their perspectives of activity from something unpleasant and difficult to something that could be enjoyable:

It helped me understand that I can move and do more activity. I can go for a walk outside and enjoy it and not have to focus on being in pain all the time. So, I think it just made me realise that that was actually an option.

Female, 21 years, European, hip pain

The participants also felt that the VR sessions improved their moods. They described feeling happier and more content following the sessions and explained that VR provided relief from daily stressors and an opportunity to do something enjoyable:

Mentality wise, it made a big difference. I looked forward to coming to the VR sessions. I’d be like—yeah, I’ve got this pain but at least something’s happening, and I have fun when I’m there.

Female, 23 years, European, pelvic pain

Overall, participants described the intervention as having a positive impact on their mood and function and reported increased confidence in participating in physical activities despite continued pain.

#### Theme 3: The Importance of a Well-Designed VR Setup

Most participants found the VR equipment easy to use and the games straightforward to understand and play. The exceptions were those whose first language was not English and who described difficulties in understanding game instructions. Participants emphasized the importance of a comfortable and adjustable headset, and the physical space was considered important, with all participants feeling constrained by the size of the room. There were a range of opinions regarding the importance of having a trained physiotherapist deliver the VR; however, all participants highlighted the importance of being supervised by someone with whom they could form a therapeutic alliance:

I think it makes you feel better that it’s a trained physiotherapist. You knew they had that background and it just fills you with confidence a bit more.

Female, 64 years, European, back pain

Overall, this theme demonstrates the importance of investing in a good-quality VR setup that participants can use easily and comfortably in a safe and supervised environment and the value of developing a strong therapeutic relationship.

## Discussion

### Principal Findings

This mixed methods pilot study assessed the acceptability and feasibility of a VR RCT in a hospital-based chronic pain treatment center. The findings indicated that the VR intervention was highly acceptable to patients, with session rating scores, treatment satisfaction, and perceived improvement all surpassing the a priori acceptability criteria. Those in the VR treatment showed improvements in pain intensity, pain interference, step counts, and activity scores, with preliminary findings suggesting that VR may be superior to no treatment and equivalent to TAU. Qualitative data supported the quantitative findings and indicated that participants enjoyed the VR treatment and found it beneficial.

Despite the benefits, poor recruitment and high dropout rates suggest that it would not be feasible to conduct an adequately powered RCT in this setting. ESs for primary outcomes in this study indicate that a reasonable sample size of approximately 60 per group (power=0.80) would be adequate to detect effects. These ESs are consistent with recommended ESs for motor interventions [34] and comparable with another VR RCT currently in progress [24]; however, the recruitment rate of approximately 2 participants per month means that an adequately powered RCT would not be feasible. In addition, any future RCT assessing multiple pain-relevant outcomes would need to carefully select the most relevant outcome measures and correct for the family-wise error rate. Attrition was higher than expected, with approximately 45% of enrolled participants withdrawing from the study. Similar rates of withdrawal across all treatment arms suggest that attrition was not specific to the treatment, with the most frequently cited reasons for withdrawal being (1) comorbid mental health problems, (2) inability to attend regular appointments, and (3) loss of contact following SARS-COV-2 lockdowns. High attrition is common in chronic pain settings [11], and future work may benefit from encouraging participation by offering treatment in community settings, ensuring adequate parking, and providing transportation and remuneration for attendance. Finally, although the findings suggest that the involvement of a qualified physiotherapist is important, remotely delivered VR therapies have shown promise [11], and future RCTs may benefit from adapting active VR treatments for home use.

### Comparison With Prior Work

Most prior studies exploring the role of VR in chronic pain have focused on specifically designed health applications [23,25], targeted singular pain sites [26], or tested the efficacy of VR among veterans with chronic pain [33]. This is the first study to assess the feasibility of conducting an RCT that tests whether commercially available active VR games may improve outcomes among people attending a tertiary-level chronic pain treatment center. Consistent with previous studies [23], high levels of engagement and treatment satisfaction were reported, and the findings suggest that active VR may be equivalent to standard physiotherapy [25,26]. When considering potential mechanisms, it has been hypothesized that VR might improve functional outcomes by reducing kinesiophobia, and prior work has documented reductions in kinesiophobia following VR interventions [26,33,52]. However, our findings did not support this hypothesis. Instead, qualitative data suggest that increases in general well-being and pain self-efficacy are more likely mechanisms. Positive mood states may be a protective factor in chronic pain [53,54], and a previous study has shown that a VR intervention among people with fibromyalgia led to improvements in general mood state, positive emotions, motivation, and self-efficacy [55]. Future RCTs may benefit from the inclusion of measures of pain self-efficacy [56] along with positive affect, sleep, and other pain-relevant quality of life measures to identify the mechanisms by which VR interventions improve chronic pain outcomes.

When considering game design and selection, commercially available games were used for this pilot study because of their benefits in terms of access, convenience, and cost. Using commercially available games in chronic pain rehabilitation also makes theoretical sense, as when pain is attributed to nociplastic mechanisms, the key interventional targets are general physical activity, stress reduction, and pain self-management [57] rather than focusing on specific movements, muscles, or joints. Despite the benefits of using specifically designed apps in some health settings [28,56], games that target specific pain sites, such as neck pain or back pain, would only be suitable for a subset of people attending chronic pain treatment centers, whereas commercially available games are more likely to be applicable to people with a range of pain conditions and pain sites and varying degrees of functional impairment. In addition, commercially available games appear to be at least equivalent to conventional therapy in diverse physical rehabilitation settings [32]. Overall, whether commercially available or specifically designed games are used, it is important that games are developed using fundamental design principles of reward, goals, challenge, and meaningful play [58], and it seems likely that combining active VR treatment with other established interventions such as pain neuroscience education, as well as strategies to downregulate autonomic arousal, may strengthen the positive effects of VR in chronic pain management and enhance our understanding of how VR can supplement conventional care.

### Limitations

This study had several limitations. Primarily, comparing active VR with a no-treatment control and physiotherapy TAU allowed for comparisons between active VR and standard physiotherapy-led activity programs but did not provide insight into the most salient elements of VR or gaming interventions. Future work administering an active non-VR control, such as computer-based games using similar movements but without a head-mounted display, or comparing active VR with passive VR treatments that facilitate downregulation of autonomic arousal, would help to clarify the unique benefits specific to immersive, embodied, and active VR protocols over and above other VR and gaming platforms. Furthermore, this study deviates from a standard 3-arm trial design as, for practical purposes (given the small pool of participants), a single group covered 2 intervention arms (waitlist and TAU). Future work would benefit from recruiting separate groups of participants for the TAU and no-treatment arms. In addition, it is likely that patients interested in VR would have self-selected for the trial and potentially overreported the perceived benefits. Another limitation is that session rating scales were not collected in the TAU arm, meaning that between-group comparisons for session enjoyment and changes in pain scores immediately after treatment could not be made and that VR and TAU were delivered at different doses, with VR offered twice weekly and TAU delivered once weekly; thus, it is not known whether VR delivered at the same dose of TAU would have produced the benefits seen here. Finally, end users were not consulted in the study design phase, and future RCTs would benefit from engaging in a co-design process.

### Conclusions

Despite these limitations, this mixed methods pilot study indicates that active VR is an acceptable treatment for patients attending a tertiary-level chronic pain treatment center. Qualitative data suggest that participants enjoyed the VR treatment, found it beneficial, and believed that it was an acceptable component of chronic pain rehabilitation. Although outcomes for VR in this pilot study were superior to no treatment, and appeared to be at least equivalent to standard physiotherapy, these findings should only be interpreted as a basis for designing future adequately powered clinical trials. In particular, the finding that the ESs comparing VR with standard treatment were generally small suggests that there may not be clinically meaningful differences between these groups in a larger trial. Despite this, an adequately powered RCT appears justified as, if equivalence is found, then VR may be a useful adjunct or alternative to standard treatment for some people with chronic pain. Although a future RCT is warranted, low recruitment and poor retention rates indicate that this would not be feasible in the present setting. Future RCTs would benefit from incentivizing study participation and actively reducing dropout rates while considering a broader range of outcome measures to identify likely mechanisms. VR technology is increasingly affordable and accessible and may improve chronic pain outcomes by encouraging participation in activities in a novel and enjoyable way. Adequately powered RCTs with long-term follow-ups examining the recommended pain-relevant outcomes and potential mechanisms of action are warranted.

## Appendix

Multimedia Appendix 1 Virtual reality software programs graded from levels 1 to 6.

## Abbreviations

BPI: Brief Pain Inventory
CASP: Critical Appraisal Skills Program
ES: effect size
MCID: minimal clinically important difference
RCT: randomized controlled trial
TARPS: The Auckland Regional Pain Service
TAU: treatment as usual
TSK-13: Tampa Scale of Kinesiophobia-13
VR: virtual reality
