# Supplementary material for: Active Virtual Reality for Chronic Primary Pain: Mixed Methods Randomized Pilot Study
Source: JMIR Form Res. 2022 Jul 13;6(7):e38366. doi: 10.2196/38366 (PMC9330488; doi:10.2196/38366)
Supplement: Multimedia Appendix 1 [file formative_v6i7e38366_app1.docx]

| *Appendix A: Virtual reality software programs graded from levels 1 to 6* | | | | | | |
| --- | --- | --- | --- | --- | --- | --- |
| *Grade* | *Game* | *Release Date* | *Genre ^a^* | *Publisher* | *Developer* | *Website* |
| 1 | Fruit Ninja VR | Jul 2016 | Action, Casual, Simulation, Sports | Halfbrick Studios Pty Ltd | Halfbrick Studios Pty Ltd | https://www.halfbrick.com/games/fruit-ninja-vr |
|  | Holodance | Feb 2020 | Casual, Indie, Sports | Narayana games UG | Narayana games UG | http://holodance-vr.com |
| 2 | Candy Smash VR | Jul 2016 | Action, Casual, Indie, Simulation, Sports | Trinity Project | Wadup Games | no dedicated website |
| 3 | QuiVR | Jun 2018 | Action, Casual, Indie, Simulation | Alvios, Inc. | Blueteak | http://quivr.net |
|  | NBA 2KVR Experience | Nov 2016 | Casual, Sports | 2K | Specular Interactive | no dedicated website |
| 4 | Lightblade VR | Jun 2016 | Action, Indie, Simulation | Andreas Hager Gaming | Andreas Hager Gaming | https://lightbladevr.mazebert.com/ |
|  | Bitslap | Sep 2016 | Indie | Comrex AG | Comrex AG | no dedicated website |
| 5 | Space Pirate Trainer | Oct 2017 | Action | I-Illusions | I-Illusions | https://spacepiratetrainerdx.com/ |
| 6 | Fancy Skiing VR | Aug 2016 | Adventure, Sports | HashVR Studio | HashVR Studio | http://www.hashvr.com/games/5 |
|  | Doritos VR Battle | Nov 2016 | Action, Casual, Indie | Capitola VR | Capitola VR | http://www.doritos.nl/ |
| ^a^ Genre category available from https://store.steampowered.com/ 2021 Valve Corporation | | | | | | |
